# Predicting Change in Depressive Symptoms in the Nursing Home

**DOI:** 10.1093/geroni/igaa057.1239

**Published:** 2020-12-16

**Authors:** Joann Reinhardt, Orah Burack, Verena Cimarolli, Audrey Weiner

**Affiliations:** 1 The New Jewish Home, New York, New York, United States; 2 LeadingAge, Washington, District of Columbia, United States

## Abstract

Depressive symptoms are common among nursing home (NH) residents. Important predictors of depression to tease apart include demographic characteristics, physical status, functional ability, and chronic pain. A challenge to addressing depression is that a majority of NH residents have some level of dementia. Nonpharmacological management of depression in the NH is a recommended first line of treatment including: personalized activities, music therapy, repositioning, and attention to personal care needs (toileting, resting, and hydration). A holistic approach to the well-being of NH residents is the adoption of person directed (PDC) care models. In this study, predictors of decreased depression over time was examined in residents (N=144) living in two communities featuring PDC models, and those living in traditional care communities within the same NH. Care in the two PDC communities focused on provision of comfort care for persons with advancing dementia at-risk of not having their care needs met largely due to their inability to clearly communicate these needs. Care practices focused on knowing each elder deeply, and anticipating their needs. Care practices also included an emphasis on staff empowerment and meaningful life activities for residents. Traditional communities are those where PDC practices had not yet been incorporated. Data on demographic characteristics, cognitive status, physical and functional status, behavioral symptoms, and pain were extracted from the MDS. Results showed that being in the PDC group, less time in the nursing home, having less pain, and fewer behavioral symptoms were significant predictors of decreased depressive symptoms over a six-month period.

